# Factors contributing to students and instructors experiencing a lack of time in college calculus

**DOI:** 10.1186/s40594-017-0070-7

**Published:** 2017-06-14

**Authors:** Jessica Ellis Hagman, Estrella Johnson, Bailey K. Fosdick

**Affiliations:** 1 Department of Mathematics, Colorado State University, 1874 Campus Delivery, Fort Collins, CO 80523-1874 USA; 2 Department of Mathematics, Virgina Tech University, 460 McBryde Hall, Virginia Tech, 225 Stanger Street, Blacksburg, VA 24061-0123 USA; 3 Department of Statistics, Colorado State University, 102 Statistics Building, Fort Collins, CO 80523-1877 USA

**Keywords:** Post secondary, Pacing and coverage, Internal and external framing, Opportunities to learn, Quantitative analysis, hierarchical linear modeling

## Abstract

**Background:**

Calculus is a foundational course for STEM-intending students yet has been shown to dissuade students from pursuing STEM degrees. In this report, we examine factors related to students and instructors reporting a lack of time in class for students to understand difficult ideas and relate this to students’ and instructors’ perceptions of opportunities to learn using a hierarchical linear model. This work is part of the US national study on college calculus, which provides an ideal landscape to examine these questions on a large scale.

**Results:**

We find a number of student factors associated with students experiencing negative opportunities to learn, such as student gender, lacking previous calculus experience, and reports of poor and non-student-centered teaching. Factors weakly associated with instructor reports of lack of time were a common final and reporting that approximately half of the students lacked the ability to succeed in the course.

**Conclusions:**

This analysis offers insight into how we might create more positive opportunities to learn in our own classrooms. This includes preparing students before they enter calculus, so they feel confident in their abilities, as well as weakening the internal framing of the course by engaging in teaching practices that provide students opportunities to communicate and influence their learning (e.g., discussion and group work). We argue that this is especially important in introductory college calculus courses that are packed with material, taught to a diverse population of students in terms of demographics, mathematical preparation, and career goals.

## Background

It is estimated that as many as 40% of science, technology, engineering, and mathematics (STEM) intending majors do not graduate with a STEM degree (Hurtado et al. 2010). This is especially problematic in the USA, where the number of STEM graduates must increase by an additional one million over current projections in the next decade to match expected workforce demands (PCAST 2012). Many of the students leaving STEM majors cite ineffective teaching methods and uninspiring atmospheres in introductory-level STEM courses—with introductory mathematics courses often singled out—as the primary reason for attrition (Niemi 2002; Seymour 2006; Thompson et al. 2007; PCAST 2012; Rasmussen and Ellis 2013). In the USA, Calculus I, a course typically including limits, the definition of the derivative, and differentiation rules and applications (see Johnson 2016 for more detail about Calculus I content), is the first mathematics course many students take when entering college. This course is required for all students intending to pursue a STEM degree and has some of the highest enrollment numbers. In the fall of 2010, the year that the data presented here were collected, there were over 234,000 students taking Calculus I across the USA (Blair et al. 2013). However, while this course is heavily populated and required for all STEM degrees, the US colleges and universities are struggling with high attrition and failure rates in calculus.

When asked why they are leaving STEM degrees, students often report being frustrated with courses overburdened with content and with pacing structures that inhibit comprehension and reflection (Seymour 2006). As Calculus I is a requirement for many diverse STEM-related disciplines, these courses typically cover a large amount of content and thus are prone to create the negative atmosphere described by students that leave STEM majors. Notably, Calculus I attrition is not uniform across demographic groups. For instance, after controlling for student preparedness, intended career goals, and perceived course instruction, women are 50% more likely to switch out of the calculus sequence compared to men (Ellis et al. 2016). These retention problems, both with STEM students in general and women and underrepresented minorities in particular, do not appear to be US specific; van Langen and Dekkers (2005) found that Sweden, the UK, the US, and the Netherlands are all struggling with enrollment and persistence with STEM degree courses. They attribute this to “a general declining interest, an underrepresentation of women, acute shortfalls on the labor market and high economic ambitions” (p. 336).

Mathematics instructors appear to be aware of these issues, and the US mathematics community has had, and continues to have, lively debates about both the breadth and depth at which topics should be addressed in Calculus I (e.g., Wu 1999; Yoshinobu and Jones 2012). In this paper, we explore what factors are associated with a person’s perception that there is enough time in class to understand the more difficult ideas of the course, first from the student perspective and then from the instructor perspective. These analyses give us insight into what factors guide perceptions of the pacing of the course—in particular, a course that is typically overstuffed with material (Seymour and Hewitt 1997).

## Methods

### Theoretical perspective

Presumably, the more content taught in a course, the more content a student can learn in a course. In this way, the amount of material covered is directly related to the students’ *opportunities to learn* (OTL)*.* However, the sheer number of topics in a course is just one facet of OTL. As summarized by Reeves et al. (2013), the OTL construct is comprised of three components: (a) content coverage (list of topics and subtopics covered), (b) content exposure (amount of time devoted to instruction and time-on-task), and (c) content emphasis (which topics are selected for emphasis). In this paper, we examine factors related to student and instructor perceptions of the abundance of opportunities to learn.

Previous research on OTL has largely focused on the factors affecting students’ exposure to opportunities to learn and their disposition or ability to take up and actualize those opportunities. Much of this has centered on the uneven distribution of opportunities to learn across demographic groups and other student populations (e.g., Flores 2007; Oakes 1990). This work has shed light on the direct connection between divergent mathematical outcomes across student populations and the unequal distribution of opportunities to learn mathematics afforded to these populations. Additionally, this works highlights the role of students’ mathematical dispositions in actualizing these opportunities to learn (e.g., Gresalfi 2009), as well as the role of different pedagogical environments in providing different amounts of and types of opportunities to learn (e.g., Yackel et al. 1991). Together, this indicates that factors stemming from students’ backgrounds, their mathematical dispositions, and their classroom experiences contribute to how they experience and actualize content coverage, exposure, and emphasis. In other words, a student’s perceptions of, and ability to capitalize on, OTLs depend on both the individual student and his/her instruction.

From instructors’ perspectives, multiple factors contribute to how they decide to structure a course in regard to content coverage, exposure, and emphasis. For instance, 99% of Calculus I students are not math majors, with 30% coming from engineering (Bressoud et al. 2013). As a result, Calculus I is primarily a service course, where the client disciplines (e.g., engineering, physics, chemistry) have a strong influence over what topics are taught. Additionally, because of the large number sections taught at a university in any given semester, Calculus I courses are often coordinated (Rasmussen and Ellis 2015). This coordination frequently includes a common syllabus, schedule, textbook, exams, and homework. These factors, all external to the student-teacher interactions within the classroom, contribute the *external framing* of the course. External framing refers to the influence of agents outside of the classroom—such as administrators, professional societies, policy documents, and parents—over various aspects of teaching, including coverage and pacing (Hoadley 2003). *Internal framing* refers to the amount of influence, or perceived influence, of the students over various aspects of teaching (Haugen 2011). Internal factors influencing content coverage, exposure, and emphasis include interactions between the instructor and the students, student preparation, or perceptions thereof, and the results and influence of formative and summative assessments.

Both external and internal framing can either be strong or weak, where the degree of framing is indicative of the degree to which different actors control, or feel they have agency to control, what happens in the class (Hoadley 2003). A strong external framing indicates that the instructor has little control over what or how she teaches. For instance, we may expect a new graduate teaching assistant who is teaching a coordinated section of calculus to have strong external framing. A weak external framing indicates that the instructor has more freedom related to content, exposure, and emphasis in the course, and thus provides the instructor more control over the OTLs she provides. A strong internal framing indicates that the teacher sets the pace and emphasis of the course, regardless of cues from the students, whereas a weak internal framing indicates that the instructor has a less ridged structure for the course, yielding some of the agency and power to the students. For instance, a classroom where the teacher regularly adjusts the lesson based on feedback from the students has weak internal framing. While this can occur during a very interactive lecture, this sort of weak framing is typical of classrooms implementing student-centered instructional approaches, as these approaches are, by design, guided by the students rather than the instructor (Haugen 2011).

External framing also has a strong influence over how much control the instructor can yield to the students (i.e., the internal framing). For instance, an instructor might be very aware that the content, exposure, or emphasis of their course is not supporting student learning, but strong external framing prohibits adjusting the OTL for her students (e.g., she does not have the option to alter the pacing in class based on feedback from students because of the external constraints). Alternatively, it may be the case that an instructor is not adjusting the OTL for her students because she is teaching in a way that inhibits her from receiving feedback from students (e.g., by not asking for students to give input in class) or not attending to this feedback (e.g., by not adjusting her teaching based on student input). In either scenario, the students would experience strong internal framing that results in the students lacking the ability to influence their OTL.

Negative OTL experiences, coupled with strong internal or external framing, are indicative of a loss of agency of either the student or the instructor. Strong external framing that mandates a strong internal framing indicates that the instructor has limited agency in providing positive OTL for her students. Similarly, strong internal framing coupled with a negative OTL experience indicates the students have reduced agency in their ability to learn. This sense of loss of student agency is of particular importance in Calculus I, as self-efficacy is a strong predictor of first-year college GPA (Zajacova et al. 2005).

Instructors are likely to feel the least amount of agency in the classroom when there is strong external framing coupled with weak internal framing—such as in a highly coordinated course being taught with a student-centered approach. Instructors are likely to feel the most agency when there is weak external framing coupled with strong internal framing—such as in a course with high instructor autonomy being taught in a rigid, lecture-based approach. Students’ perceptions of agency in the classroom are likely less based on external framing; their perception of having the most agency in the classroom will likely occur with weak internal framing, regardless if this is coupled with strong or weak external framing as they are often unaware of external factors. If the course is taught in a way that does not respond to students’ needs, the students will likely feel that they have little control over the OTL in class, whether this be because of a packed and rigid common curriculum or because of the individual instructor’s decision to teach in such a way. Thus, we would expect that students will feel that they did not have enough time to understand difficult ideas in classes where students have the least agency in the classroom, i.e., in classes with strong internal framing. Similarly, we would expect instructors to feel that there was not enough time for their students to understand difficult ideas when there is strong external framing.

For this paper, we seek to understand what factors influence OTL pacing of calculus instruction, and how these influences are related to internal and external framing. Specifically, we investigate the following research questions:

1. What are the characteristics of students who reported a positive versus negative OTL experience in Calculus I?
2. What are the characteristics of instructors who perceived their students were experiencing a positive versus negative OTL?

### Project background and data collection

Data for this project comes from the Characteristics of Successful Programs in College Calculus (CSPCC) project.^1^ CSPCC is a national study designed to investigate Calculus I in the USA, and in the fall of 2010 involved online-surveys sent to a stratified random sample of Calculus I coordinators (or department chairs). These coordinators sent the survey to instructors who were currently teaching Calculus I, and those instructors sent the survey to their students. The students and instructors were asked to complete both a start-of-term and end-of-term survey. For the data analyses we are presenting here, we have complete data (related to all questions in these analyses) for 2562 students and 327 instructors from 127 institutions. Further, this data is linked, meaning that we can match students to instructors and institutions.

### Measuring perceptions of OTL

The outcomes we consider in this study are student and instructor reports of OTL. We use the following questions as proxies for OTL: on the student survey, “My Calculus instructor allowed time for me to understand difficult ideas” with answer options (1) strongly disagree, (2) disagree, (3) slightly disagree, (4) slightly agree, (5) agree, and (6) strongly agree; on the instructor survey, “When teaching my Calculus class, I had enough time to help students to understand difficult ideas” with answer options (1) never, (2) infrequently, (3) frequently, and (4) very frequently. For the student survey, answers 1–3 were grouped together to indicate that the students reported that there was not enough time in class for them to understand difficult ideas, and answers 4–6 were grouped together to indicate that the students reported that there was enough time. Similarly, on the instructor survey, answers 1–2 were grouped together for “not enough time” and 3–4 were grouped together for “enough time.”

What does it mean to report that you do not have enough time to understand difficult ideas? There are three components embedded within this question: what it means to have enough time, what it means to understand, and what are difficult ideas. In this paper, we are interested in student’s ability to capitalize upon the OTLs present in class. Therefore, we are most interested in attending to whether or not students thought there was enough time for them to learn what was presented in the course. However, students in the same class may respond differently to this question not only because they perceived the pacing differently but also because they have different perspectives on what it means to understand an idea or different perspectives on what ideas are difficult. Thus, reporting that there was not enough time may indicate a number of things, such as the instructor’s teaching style and/or pacing did not help the student to understand difficult ideas; the coordinated exams did not align with the OTLs provided in class; the student was not prepared for class and so, perhaps regardless of instruction, did not feel that there was enough time to understand difficult ideas; or the student may not believe that she was capable of understanding the difficult ideas of calculus.

Similarly, from the instructor’s perspective not having enough time to help students understand difficult ideas may be related to a variety of things, such as the instructor felt that she had to teach faster than she would have preferred because of external framing, or that the students were not capable of learning the material presented in the amount of time available (and perhaps may not be capable of learning the material regardless of the amount of time available).

### Factors potentially related to perceptions of OTL

Student and instructor reports of negative OTL (i.e., that there was not enough time for students to understand difficult ideas) may be associated with factors related to strong framing, but it may also be associated with factors not directly related to framing, including student preparation, beliefs about student ability, and beliefs about what it means to understand difficult ideas. To begin to disentangle the multitude of factors related to the pace of a course as well as perceptions on the pace of the classroom, we conduct two separate regression analyses: one to understand what factors predict students’ perceptions of whether there was enough time to understand difficult ideas and one to predict instructors’ perceptions of whether there was enough time. For both analyses, we use the binary responses to the “enough time for difficult ideas” question as the outcome and consider a number of questions from the student and instructor beginning- and end-of-term surveys related to the potential explanatory factors. In this section, we describe the factors that we explore related to student and instructor reports of OTL and provide rationale for their inclusion in this study. In Table 1, we summarize the factors hypothesized to be related to perceptions of pacing and the survey questions used to investigate each factor for both students and instructors. All of the survey items analyzed can be found in Appendix A.

**Table 1:** Factors and variables used in student and instructor analyses

Factor	Student analysis	Instructor analysis
Traditionally marginalized or vulnerable populations	• Gender	• Gender • Instructor rank (GTA, lecturer, tenure-track faculty, tenured faculty)
Classroom features	• Student reports of “student-centered practices”	• Common final • Instructor reports of “student-centered practices”
Student preparation and perception of student preparation	• Previous calculus experience (none, high school, college) • SAT/ACT mathematics percentile • Ability to succeed: “I believe I have the knowledge and ability to succeed in this course” (agree versus disagree)	• Perceived student ability: “Approximately what percentage of students currently enrolled in your Calculus I course do you expect are academically prepared for the course?” (>80, 60–80, 40–60, or <40%)
What it means to “succeed” in calculus	• Success perception: “My success PRIMARILY relies on my ability to” (“solve specific kinds of problems” versus “make connections & form logical arguments”)	• Success perception: “From your perspective, student’s success PRIMARILY relies on their ability to” (“solve specific kinds of problems” versus “make connections and form logical arguments”)
Grouping factor	• Instructor	• Institution

The first set of factors we investigate in the student and instructor analyses are demographics related to traditionally marginalized or vulnerable populations. One may expect that these populations would have less agency and influence over what happens in class, so that marginalized or vulnerable populations would report negative OTL experiences. For students, we attend to gender because multiple studies indicate that women are more likely to leave their STEM intentions after their experiences in STEM courses such as Calculus I (Ellis et al. 2016; Seymour and Hewitt 1997). We do not investigate the association between race or ethnicity and OTLs due to the small proportion of non-white students and instructors in our study. For instructors, we also include gender because studies indicate women are less represented in mathematics departments and may have less agency related to their instruction (Hill et al. 2010). Additionally, we consider different instructor ranks (GTAs, lecturers, tenure-track, and tenured) since certain types of instructors may be more likely to have greater external framing and thus more instances of negative OTL (for instance, Rasmussen and Ellis (2015) found that faculty are able to opt out of most of the coordinated components of calculus instruction at many institutions).

The second set of factors we consider involves classroom features. At the student level, we include one aggregate variable characterizing student reports of student-centered practices (e.g., holding whole class discussions, having students work together in groups, and having students present their work to the whole class). We include student reports of instruction to understand how student perception of what the instructor did in class is related to their perception of OTL. One may expect that reports of fewer student-centered practices would be related to strong internal framing, and thus negative OTL experiences. We also include *instructor* in the student analysis to control for variation among student OTL responses due to the individual instructors, rather than measured aspects of the class related to the instructor, such as the instruction methods. In other words, we cluster students by instructor to control for potential additional unmeasured characteristics of the instructor that are related to student perception of OTL. To investigate classroom features at the instructor level, we consider instructor reports of student-centered practices, a variable that parallels the student reports of student-centered practices, and the existence of a common final exam. We include these instructor reports of instruction to understand how instructor perception of what he or she did in class is related to their perception of OTL. We also include existence of a common final exam as an indicator of external framing from the department, as it represents a set expectation of coverage. Finally, we include *institution* to control for variation among instructor responses about OTL due to unmeasured aspects of external framing. This amounts to clustering instructors by institution in the analysis to control for potential additional unmeasured characteristics of the Calculus I course structure at institutions that is related to instructor perception of OTL.

The third set of factors we consider is student preparation and perception of student ability. For the student analysis, we include previous calculus experience, standardized math test percentile, and whether the student agreed with the statement “I believe I have the knowledge and ability to succeed in this course.” The first two variables characterize actual student preparation. One may expect that less preparation would be related to negative OTL because if students are not prepared, they may be less able to actualize OTLs. The third variable is related to students’ perception of their ability to actualize OTLs. It has been found that student self-efficacy is connected to mathematical achievement (Wach et al. 2015) and career goals (Ellis et al. 2016; Lent et al. 1994). For the instructor analysis, we consider instructors’ perception of their students’ ability. Instructors were asked to report, “Approximately what percentage of students currently enrolled in your Calculus I course do you expect are academically prepared for the course?” with options of more than 80, 60–80, 40–60%, and less than 40%. One would expect that instructors who report a lower percentage of expected student success would also report negative experiences with OTL.

The final set of factors that we investigate relates to what it means to succeed in calculus. Both students and instructors were asked if student success in calculus is more reliant on students’ ability to solve specific kinds of problems or more reliant on students’ ability to make connections and form logical arguments. These questions speak to students’ and instructors’ view of what it means to understand “difficult ideas,” and thus, their perceptions of what it means to actualize OTLs in calculus. For instance, instructors and students may conceptualize “difficult ideas” in Calculus I as those ideas related to solving difficult problems, such as differentiating a complicated function or as ideas related to making difficult connections, such as how differentiation is related to integration beyond the procedural relationship. This item also provides insights into their perceptions of success as more related to performance goals or learning goals (Dweck 1986). If a student or instructor responds that student success in calculus is more related to solving specific kind of problems, this could be indicative of a perspective on learning more closely related to performance goals: learning with the goals of increasing one’s ability to demonstrate high competence. On the other hand, if a student or instructor responds that student success is more related to one’s ability to make connections and form logical arguments, this could be indicative of a perspective on learning more closely related to learning goals: learning with the goal of increasing one’s competence. Research has shown deep connections between a learning goal perspective and higher rates of persistence and achievement (Spinath and Stiensmeier-Pelster 2003).

### Summary of survey responses

In this section, we provide a brief overview of the student and instructor responses on the above factors (tables for this data can be found in Appendix B). In our data set of over 2500 students, around half were male and over two-thirds had taken calculus in high school or college. Of the over 300 instructors in our data set, nearly three-quarters were male and roughly a third were lectures, a third were tenured faculty, and the remaining were GTAs or tenure-track faculty. Over 80% of the students reported that they felt prepared for the course while less than a third of faculty reported that they believed over 80% of their students were prepared. When asked if student success in calculus relies more on solving specific kinds of problems or making logical connections, nearly 80% of students and over 60% of instructors said solving specific kinds of problems. Finally, 77% of students and 70% of instructors agreed that there was enough time in class for students to understand difficult ideas.

### Regression analyses

The outcome variables used in the analyses serve as a proxy for student and instructor perception of OTLs. The outcome in the student analysis was whether each student responded affirmatively or negatively to the statement, “My Calculus instructor allowed time for me to understand difficult ideas.” As described in the measuring perceptions of OTL section above, original student responses to this statement on the survey were dichotomized, such that a “1” indicates a student felt there was adequate time and a “0” denotes the student did not feel there was enough time. Since this outcome is binary, we used a logistic regression model to quantify the effect of each of the variables in the second column of Table 1 on the probability that a student felt there was enough time.

There were seven explanatory variables in the student regression model derived from the variables in Table 1. Linear effects were included for student standardized test score and reports of student-centered practices. The regression coefficients on these two variables summarize the average changes in the log odds of a student reporting there was enough for a one-unit change in the explanatory variable (e.g., an increase of one percentile in a student’s standardized test score). Gender, success perception, and ability to succeed are dichotomized explanatory variables. To include them in the model, one level of each was selected as the baseline and a binary indicator variable for the non-baseline level was constructed. The regression coefficient corresponding to these binary variables then represents the effect of a student reporting the non-baseline level on the probability that the student reported enough time. Males were specified as the baseline for gender, disagreeing with the statement “I believe I have the knowledge and ability to succeed in this course” was the baseline for the ability to succeed variable, and the response “My success PRIMARILY relies on my ability to solve specific kinds of problems” was the baseline level for the success perception variable. This means, for example, the regression coefficient on gender indicates the effect of a student being female (versus being a male) on the probability the student reports feeling there was enough time in class, assuming all other variables are equal. Finally, previous calculus experience is a categorical variable with three levels. One level was selected as a baseline and two new variables were created for the regression model---one for each of the non-baseline levels. High school calculus experience was selected as the baseline category, and two new binary indicator variables were created for the regression model corresponding to having no previous calculus experience and having college calculus experience. We summarize the relative importance of each of the factors using the estimated odds ratios from the logistic regression. We report 90 and 95% confidence intervals for the odds ratios as well and assess the statistical significance of our results.^2^ See Appendix C for details on the model estimation procedure.

Finally, based on the structure of courses at universities and colleges, we expect that responses from students that have the same instructor will be correlated with one another. To account for this dependence, we include a random effect in the regression model for instructor, which aims to capture the variability in student responses not attributable to the variables in Table 1, but which may be the result of students having the same instructor. Including a random effect for instructor makes the logistic regression model a hierarchical model as it clusters students together that shared an instructor. The details of a sensitivity analysis illustrating the importance of clustering by instructor are included in Appendix C.

The regression model for the instructor analysis was constructed similarly to that for the students. A logistic regression model was used to model whether instructors responded affirmatively or negatively to the statement, “When teaching my Calculus class, I had enough time to help students to understand difficult ideas.” A binary variable was created based on survey responses where “1” indicates an instructor felt there was enough time and “0” indicates feeling there was no enough time. Ten explanatory variables were constructed for the model based on the instructor-level variables in the third column of Table 1. A linear effect was included for reports of student-centered practices. Gender, success perception, and a common final were dichotomized variables, so, again, baseline categories were selected and new indicator variables constructed. Males were again selected as the baseline for gender and “solve specific kinds of problems” was selected as the baseline for success perception. Not having a common final was also specified as the baseline level, so new indicator variables were constructed for females, believing that success depends on an ability to “make connections and form logical arguments” and having a common final exam. Instructor type and perceived student ability were treated as categorical, each having four levels. Tenure-track faculty was treated as the baseline for instructor type and 80–100% of students being prepared for the course was the baseline for perceived student ability. Indicator variables were constructed for each of the other three levels of the variables (e.g., indicators were created for tenured faculty, lecturers, and GTAs for the instructor variable).

Since mathematics department cultures and calculus programs vary across institutions, it is expected that instructors at the same institution may feel similarly with regard to whether there is enough time for students to understand difficult ideas. To account for this correlation among instructor responses, we included a random effect in the regression model for institution. This serves to capture unobserved institution-level variation in instructor responses that is not explained by the other explanatory variables included in the instructor model. Including a random effect for institution in the instructor analysis makes the logistic regression model a hierarchical model as it clusters instructors together that are at the same institution.

## Results

### Student analysis

**Fig. 1:**
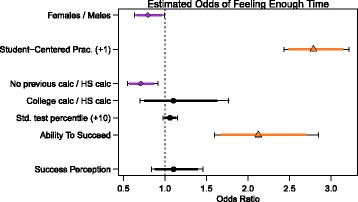
Student results. Estimated effects of the student factors on the odds that a student reported there was enough time in class to understand difficult ideas. The *points* represent the best estimate of the odds ratio, and the intervals represent the 90% (smaller, colored or *black*) and 95% (wider, *black*) confidence intervals for the ratios. Statistically significant odds ratios greater than one are shown in *orange with triangle points* and indicate students with higher levels of this factor are more likely to respond that there was enough time. Similarly, odds ratios in *purple with diamond points* are associated with students being less likely to respond that there was enough time

Figure 1 shows the estimated odd ratios for students reporting positive OTL. As illustrated by the purple markings and diamond points, two variables are associated with a lesser chance of reporting positive OTL: identifying as a woman versus identifying as a man^3^ and having no previous calculus experience versus having had taken calculus in high school. This means that students who are women and students who have not taken calculus before are more likely to report negative OTL compared to men and compared to students who took calculus in high school, respectively. These two factors, independent of the framing of the course, appear to impact students’ abilities to benefit from the OTLs presented in class as fully as other students may be able to.

As illustrated by the orange markings and triangle points, two variables are associated with higher reports of positive OTL: reporting more student-centered practices and agreeing with the statement “I believe I have the knowledge and ability to succeed in this course.” These two factors speak to very different reasons why a student would feel that there was enough time for them to learn the more difficult ideas in calculus––one is indicative of the classroom structure (i.e., weak internal framing) and one is student-specific. Of all factors investigated in this study, that most strongly associated with positive OTL is student-centered practices. This suggests that the biggest contributor to students reporting positive OTL is a class environment that provides them a variety of ways to learn, including whole-class discussion, opportunities to explain their thinking, and asking questions. These instructional practices all may provide students with mechanisms to influence their own learning experience, which is indicative of a weak internal framing.

In addition to student-centered practices, the belief in one’s own ability to be successful was found to be significantly associated with positive OTL, echoing previous studies that indicate having confidence in one’s ability can significantly impact one’s actual ability to succeed (Eronen et al. 1998). However, unlike the nature of the instruction, believing in one’s own ability to succeed is independent of the internal framing of the course. This analysis indicates, then, that regardless of the instructional approach, a student’s belief in their ability to learn is associated with their ability to recognize (and likely capitalize) on the opportunities to learn made available in class. Taken together with the previous results, we begin to get a better picture of how these variables may interact to affect students’ calculus experiences related to OTL. For instance, men confident in their ability to succeed who have previously taken a calculus course may be able to actualize the OTL presented to them in a course with either strong or weak internal or external framing. This may not be the case for all students; especially those coming from traditionally marginalized groups. In fact, Ellis et al. (2014) found that different populations, within the same class, reported different pedagogical experiences.

After accounting for gender, reports of instruction and previous calculus experience, three factors were not found to be significantly associated with reports of positive OTL: previously taking calculus in high school as opposed to taking calculus in college, standardized test percentile, and perception of what it means to be successful in Calculus I. Thus, these factors were either not individually as related to OTL as we hypothesized, we were unable to detect the impact of these factors based on our sample size, or after accounting for the other factors they were no longer impactful. One factor that we did not include in this analysis, but that is most certainly linked to student reports of having enough time to learn difficult ideas, is a more general perception of instruction quality. If students feel that after taking a class, there was enough time for them to learn the difficult material, then they likely also feel that the instruction was good. Indeed, the question on the student survey about having enough time to understand difficult ideas was asked in conjunction with seven other questions related to the quality of instruction, including whether the instructor provided explanations that were understandable, listened carefully to students’ questions and comments, and was available for appointments outside of office hours (see BLIND for more information). In a previous analysis drawing from the same data set but investigating persistence in STEM, these eight questions were found to be highly correlated and an aggregate variable called “Instructor Quality” was created (BLIND cite). The fact that having enough time to understand difficult ideas is correlated to other aspects of basic instructional quality indicates that providing time in class for students to learn the material is a component of and related to good teaching overall.

### Instructor analysis

**Fig. 2:**
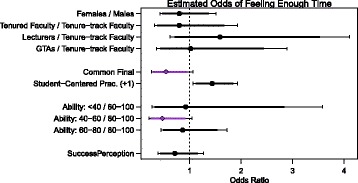
Instructor results. Estimated effects of the instructor factors on the odds that an instructor reported there was enough time in class to understand difficult ideas. The *points* represent the best estimate of the odds ratio, and the intervals represent the 90% (smaller, potentially colored) and 95% (wider, *black*) confidence intervals for the ratios. Confidence intervals that do not overlap one (shown in *purple with diamond points*) indicate the factor has a statistically significant association with instructor OTL reports at the .10 significance level

Figure 2 depicts the estimated odds ratios, and corresponding confidence intervals, for the factors considered in the analysis of instructors reporting that there was enough time in class for students to understand difficult ideas. Only two variables were found to be associated with instructors reporting negative OTL from the set of factors we investigated, and these two were only weakly associated (at the .10 significance level): teaching a course with a common final exam and reporting that 40–60% of students are prepared for the course compared to reporting 80–100% of students are prepared. This suggests that having a common final exam, which is indicative of strong external framing, corresponds to a decrease in the amount of OTL instructors feel they are able to provide for their students. Additionally, when instructors believe their class is roughly evenly divided between prepared and underprepared students, they are more likely to report negative OTL, perhaps because they feel less able to teach in a way so that *all* students can have enough time to understand difficult ideas. It is interesting that this result does not hold for instructors who believe less than 40% of students are underprepared; perhaps some of these instructors alter their teaching to accommodate the large fraction of underprepared students so the material is accessible to all, while others, possibly experiencing external framing, do not alter their behavior and report negative OTL. This suggests that the difficulty in providing positive OTL does not arise from the instructor’s perspective when they are teaching underprepared students, but rather when they are teaching an even combination of prepared and underprepared students. Given that roughly two-thirds of all college Calculus I students across the country have already taken a course called calculus, this finding suggests that the placement of students into classes with others of similar preparation may help to allow instructors to provide opportunities to learn for all students in their class.

While student-centered practices were a significant factor for positive OTL for students, it was not significant for instructors. When considering this variable, we had posited that the use of student-centered teaching practices could result in weak internal framing, where the teacher has less agency in the classroom, and a feeling that they did not have enough control to ensure OTL. Indeed, a prevalent argument against implementing student-centered pedagogy is that doing so would come at the cost of coverage (e.g., Johnson et al. 2013; Niemi 2002; Roth McDuffie and Graeber 2003; Wagner et al. 2007; Wu 1999). We did not find significant evidence for this argument in this study. This may be the result of the sample size, the model considered, or more substantial implications about the veracity of the argument.

Our difficulty in modeling the instructors’ perceptions of OTL (e.g., only being able to identify two factors when considering a 0.10 significance level) calls for more research in this area. There are a number of possible explanations for why the research-informed factors that we investigated failed to be significant in our model. For example, this may indicate the survey questions analyzed did not adequately measure what we anticipated or that our sample was too small to identify existing relationships between the factors and OTL. Alternatively, it could be that the factors investigated are not associated with instructor perception of OTL. If this is the case, we are faced with a pressing follow-up question: If being part of a marginalized or vulnerable population, perceiving negative student preparation, classroom features, and perspectives on success in calculus are not associated with negative OTL (from the instructor’s perspective), then what is?

## Discussion and conclusions

In this study, we set out to investigate factors related to student and instructor perceptions of the pacing of Calculus I, specifically related to the amount of opportunities to learn the more difficult ideas of calculus that were provided in the course. Calculus is a service course for almost all science, technology, engineering, and mathematics (STEM) degrees, and thus must support the content needs of those fields. Many students, then, experience a course that may cover a large amount of material, but do not feel that the class was set up in a way for them to actually learn the material, resulting in many students abandoning their STEM interests altogether (Seymour and Hewitt 1997).

To better understand student and instructor perceptions of the opportunities to learn difficult material in their calculus classes, we explore factors related to both the internal and external framing of the course. Since introductory calculus serves many client disciplines and is taught to a large number of students, there are often significant external factors that may guide (or dictate) what material is covered in class and how. From the instructors’ perspective, there are also factors that are more internal to the classroom that guide coverage, such as the instructor’s view of the students he or she is teaching. For instructors, teaching a course with a common final and teaching to a very mixed group of perceived student ability levels both negatively impact instructors’ perception of their ability to provide OTLs to all students. We also explored what factors relate to the students’ perceptions of the opportunities to learn provided in class. Our analyses indicate that for the students, being male, having had high school calculus (compared to no previous calculus experience), a belief that they can succeed in mathematics, and more student-centered instruction are all related to positive OTL experience.

Overall, this analysis offers insight into how we might create more positive OTL in our own classrooms. This includes preparing students before they enter calculus and placing them into courses where they are most likely to succeed, so they feel confident in their abilities, as well as weakening the internal framing of the course by engaging in teaching practices that provide students opportunities to communicate and influence their learning (e.g., discussion and group work). We argue that this is especially important in introductory college calculus courses that are packed with material and are taught to a diverse population of students in terms of demographics, mathematical preparation, and career goals.

Along with these recommendations for practice, our paper offers insights for researchers. Our analyses show promise for the linkage between opportunities to learn and internal and external framing, and help to situate the construct of opportunities to learn within the broader mathematical, departmental, and institutional environments. We believe such a connection could be further explored in a number of ways. First, qualitative measures could be employed to better understand the nuanced connections, especially from the instructor’s perspective, between factors both internal and external to their classroom environment and instructors’ decisions related to pacing and coverage. Second, since our study relied on extant survey data that was not originally collection with our research questions in mind, it is possible that more targeted survey questions could add further insight into the relationships between student and instructor perceptions of opportunities to learn, and how each of these is related to various internal and external framings. Lastly, while calculus provided an ideal setting to explore our questions of interest due to the large enrollment and wide array of fields of study that are supported by calculus, calculus is not unique in these regards. It would be a worthwhile endeavor for researchers to explore how OTL and internal and external framing are related in other college content areas, including other introductory STEM courses, and in elementary and secondary mathematics.

## Figures and Tables

**Fig. 3:**
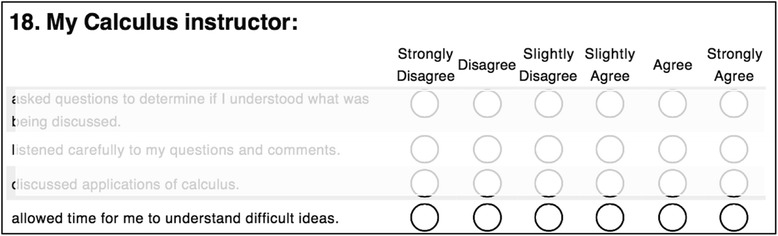
Student output variable. Responses were binned as “disagree” ={strongly disagree, disagree, slightly disagree} and “agree” ={slightly agree, agree, strongly agree}

**Table 2:**
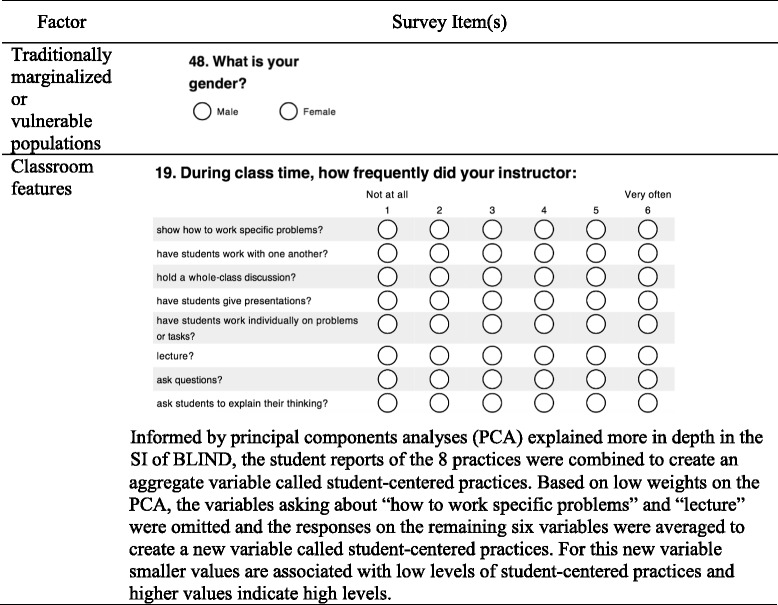
Student input variables

**Fig. 4:**
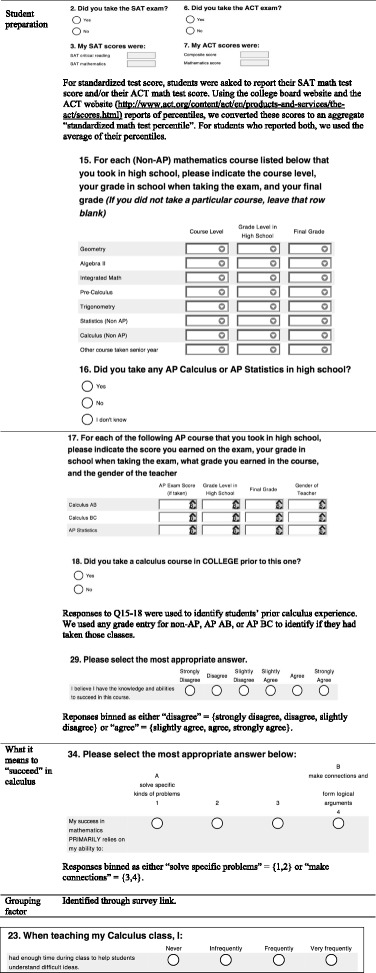
Instructor output variable. Responses were binned as “disagree” ={strongly disagree, disagree, slightly disagree} and “agree” ={slightly agree, agree, strongly agree}

**Table 3:**
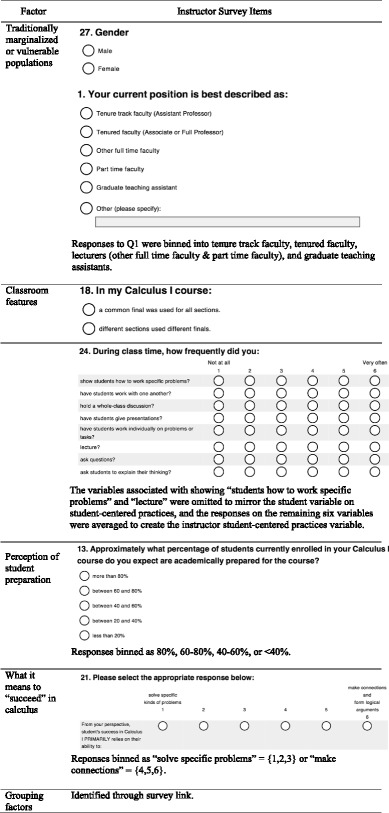
Instructor input variables

**Fig. 5:**
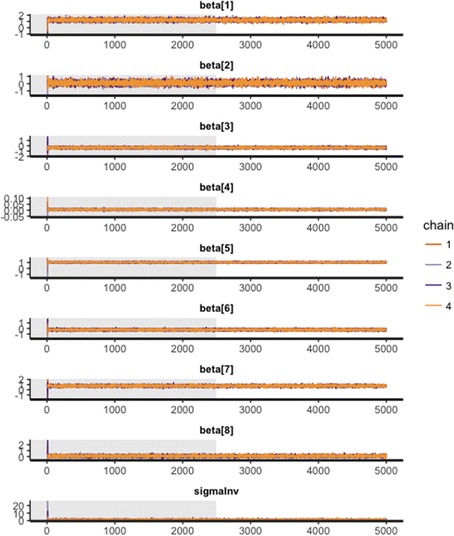
Trace plots for the student analysis. The beta parameters (1–8) correspond to the regression coefficient of the intercept, college calculus, no previous calculus, standardized test percentile, reports of student-centered practices, females, ability to succeed, and success perception, respectively. The *grayed iterations* indicate those iterations that were removed from the analysis for burn-in. None of the plots suggest problems with convergence of the Markov chain

**Fig. 6:**
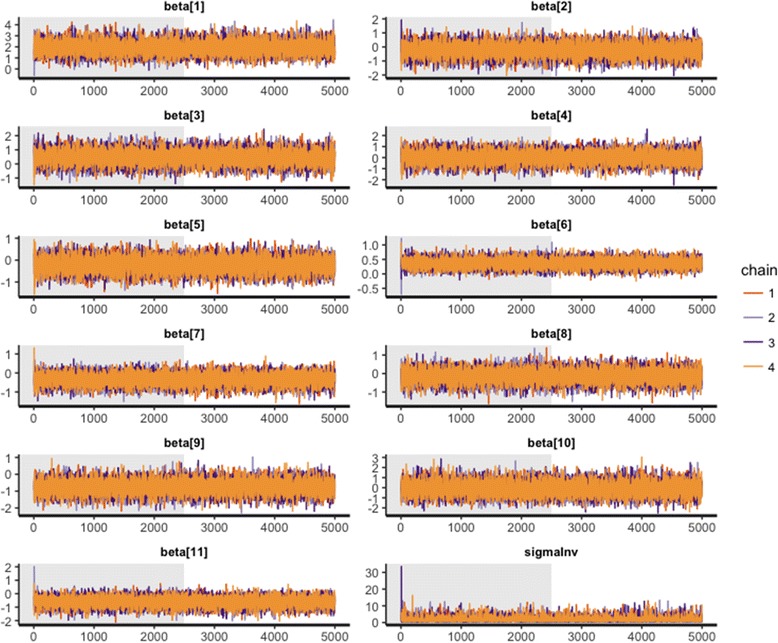
Trace plots for the instructor analysis. The beta parameters (1–11) correspond to the regression coefficient of the intercept, tenured faculty, lecturers, GTAs, females, reports of student-centered practices, success perception, 60–80% of students prepared, 40–60% of students prepared, and <40% of students prepared, respectively. The *grayed iterations* indicate those iterations that were removed from the analysis for burn-in. None of the plots suggest problems with convergence of the Markov chain

## Appendix B

### Summary data

**Table 4:** Student summary data

Factor	Variable	Responses	(*n* = 2562)
Traditionally marginalized or vulnerable populations	Gender	Male	1341 (52%)
		Female	1221 (48%)
Classroom features	Instructor reports of “student-centered practices”	1=less student-centered practices; 6=more	3.2 (1.1) (mean (std. dev.))
Student preparation and perception of student preparation	Previous calculus experience	None	764 (30%)
		High school	1610 (63%)
		College	188 (7%)
	SAT/ACT mathematics percentile		85.5 (14.2) (mean (std. dev.))
	Ability to succeed: “I believe I have the knowledge and ability to succeed in this course”	Agree	2164 (84.5%)
		Disagree	398 (15.5%)
What it means to “succeed” in calculus	Success perception: “My success PRIMARILY relies on my ability to:”	“Solve specific kinds of problems”	2023 (79%)
		“Make connections and form logical arguments”	539 (21%)
Outcome	Perception of OTL: “My calculus instructor allowed time for me to understand difficult ideas.”	Agree	1984 (77%)
		Disagree	578 (23%)

**Table 5:** Instructor summary data

Factor	Variable	Responses	(*n* = 327)
Traditionally marginalized or vulnerable populations	Gender	Male	232 (71%)
		Female	95 (29%)
	Instructor rank	GTA	61 (19%)
		Lecturer	114 (35%)
		Tenure-track faculty	43 (13%)
		Tenured faculty	109 (33%)
Classroom features	Common final	Yes	222 (68%)
		No	105 (32%)
	Instructor reports of “student-centered practices”	1=less student-centered practices; 6=more	3.3 (.98) (Mean (std. dev.))
Student preparation and perception of student preparation	Perceived student ability: “Approximately what percentage of students currently enrolled in your Calculus I course do you expect are academically prepared for the course?”	>80%	94 (29%)
		60–80%	143 (44%)
		40–60%	68 (21%)
		<40%	22 (7%)
What it means to “succeed” in calculus	Success perception: “From your perspective, student’s success PRIMARILY relies on their ability to:”	“Solve specific kinds of problems”	210 (64%)
		“Make connections and form logical arguments”	117 (36%)
Outcome	Perception of OTL: “When teaching my Calculus class, I had enough time during class to help students understand difficult ideas.”	Agree	232 (71%)
		Disagree	95 (29%)

## Appendix C

### Statistical analyses

The logistic regression models used in the student analysis and instructor analysis were each fit using a Bayesian Markov chain Monte Carlo estimation procedure with the package RStan (Stan Development Team 2016) in the statistical program R (R Core Team 2016). The prior distributions on the random effects for instructor and institution, respectively, were mean-zero normal distributions with a common variance. This is a standard prior distribution for random effects and acts to shrink the effects toward zero in the event there is not strong evidence in the data that there is substantial correlation among responses not accounted for by the explanatory variables (e.g., among responses from students taught by the same instructor). The hyperprior on the variances was an inverse gamma distribution. This prior form was chosen for convenience, as it is semi-conjugate. In addition, the shape and rate parameters of the inverse gamma distribution were set to 0.5, so the distribution would be relatively diffuse. The prior distributions on the regression coefficients were independent mean-zero normal distributions with variance equal to ten thousand. Although there is variability in scales of the explanatory variables, the large variance of this normal distribution results in a prior that is extremely diffuse and effectively allows the data alone to define the posterior distribution of the regression coefficients.

Four Markov chains were each run for 5000 iterations for each analysis, and the first half of the samples from each chain were discarded as burn-in. We assessed convergence of the chains by evaluating trace plots, which are shown in Figures 5 and 6 and did not find any evidence suggesting the chains did not converge. In addition, we considered the effective sample size of each parameter and found they were all greater than 4905 in the student analysis and greater than 8369 in the instructor analysis (see Gelman et al. 2014 for details on these standard diagnostic tools for assessing the quality of MCMC mixing). Code for the analysis is available upon request.

To investigate the impact of clustering students by instructor in the student analysis (i.e., including instructor random effects) and clustering by institution in the instructor analysis (i.e., including institution random effects), we performed sensitivity analyses fitting the logistic regression models without the respective random effects. Specifically, we looked at the squared error between the fitted probability of each student feeling there was enough time and that students response, based on the regression model with and without clustering by instructor. When instructor is included in the model, the mean squared error (MSE) is 0.123 and when instructor is not included in the model, the MSE is 0.152. Thus, we see almost a 20% reduction in error when we account for correlation among students having a common instructor. Similarly, in the instructor analysis, we considered the squared error between the fitted probability of each instructor feeling there was enough time and that instructors response, both for the logistic regression model with institution random effects and that without. The MSE when not accounting for institution is 0.195 and when clustering instructors by institution is 0.157, corresponding again to a decrease in error of almost 20%. These results suggest that there is considerable correlation among students with the same instructor and instructors in the same institution that is not accounted for by the other variables included in the model. Including the random effects in each of the models was therefore critical for obtaining accurate inference on the importance of the explanatory variables on student and instructor pacing perceptions.

## Footnotes

1 This data is available upon request at www.maa.org/cspcc.

2 We refer to findings significant at the .05 significance level as “significant” and refer to those significant at the .10 level as “weakly significant” and “weakly associated” with OTL.

3 When surveying students and instructors about gender, the survey provided options of male versus female, which address sex and not gender. However, we interpret the questions as reports of gender.

## References

[CR1] Blair R, Kirkman EE, Maxwell JW (2013). Statistical abstract of undergraduate programs in the mathematical sciences in the United States.

[CR2] Bressoud D, Carlson M, Mesa V, Rasmussen C (2013). The calculus student: insights from the mathematical association of America national study. International Journal of Mathematical Education in Science and Technology.

[CR3] Dweck CS (1986). Motivational processes affecting learning. American psychologist.

[CR4] Ellis J, Kelton ML, Rasmussen C (2014). Student perceptions of pedagogy and associated persistence in calculus. ZDM.

[CR5] Ellis J, Fosdick BK, Rasmussen C (2016). Women 1.5 times more likely to leave STEM pipeline after calculus compared to men: lack of mathematical confidence a potential culprit. PLoS ONE.

[CR6] Eronen S, Nurmi JE, Salmela-Aro K (1998). Optimistic, defensive-pessimistic, impulsive and self-handicapping strategies in university environments. Learning and Instruction.

[CR7] Flores A (2007). Examining disparities in mathematics education: achievement gap or opportunity gap?. The High School Journal.

[CR8] Gelman, A., Carlin, J. B., Stern, H. S., & Rubin, D. B. (2014). Bayesian data analysis (Vol. 2). Boca Raton: Chapman & Hall/CRC.

[CR9] Gresalfi MS (2009). Taking up opportunities to learn: constructing dispositions in mathematics classrooms. The Journal of the Learning Sciences.

[CR10] Haugen CR (2011). Educational equity in Spain and Norway: a comparative analysis of two OECD country notes. Educational Policy.

[CR11] Hill, C., Corbett, C., & St Rose, A. (2010). Why so few? Women in science, technology, engineering, and mathematics. Washington, DC: American Association of University Women.

[CR12] Hoadley U (2003). Time to learn: pacing and the external framing of teachers’ work. Journal of education for teaching: international research and pedagogy.

[CR13] Hurtado, S., Eagan, M. K., & Chang, M. (2010). Degrees of success: bachelor’s degree completion rates among initial STEM majors. Retrieved from. https://www.heri.ucla.edu/nih/downloads/2010%20-%20Hurtado,%20Eagan,%20Chang%20-%20Degrees%20of%20Success.pdf. Accessed 1 Sept 2015.

[CR14] Johnson E (2016). What is in calculus I?. MAA Focus.

[CR15] Johnson E, Caughman J, Fredericks J, Gibson L (2013). Implementing inquiry-oriented curriculum: from the mathematicians’ perspective. The Journal of Mathematical Behavior.

[CR16] Langen AV, Dekkers H (2005). Cross-national differences in participating in tertiary science, technology, engineering and mathematics education. Comparative Education.

[CR17] Lent RW, Brown SD, Hackett G (1994). Toward a unifying social cognitive theory of career and academic interest, choice, and performance. Journal of vocational behavior.

[CR18] Niemi H (2002). Active learning—a cultural change needed in teacher education and schools. Teaching and teacher education.

[CR19] Oakes J (1990). Opportunities, achievement and choice: women and minority students in science and mathematics. Review of Research in Education.

[CR20] President’s Council of Advisors on Science and Technology (PCAST) (2012). Who is switching out of calculus and why?.

[CR21] R Core Team (2016). R: a language and environment for statistical computing.

[CR22] Rasmussen C, Ellis J, Lindmeier AM, Heinze A (2013). Who is switching out of calculus and why?. Proceedings of the 37th Conference of the International Group for the Psychology of Mathematics Education.

[CR23] Rasmussen C, Ellis J, Bressoud D, Mesa V, Rasmussen C (2015). Calculus coordination at PhD-granting universities: more than just using the same syllabus, textbook, and final exam. Making the connection: research and teaching in undergraduate mathematics education.

[CR24] Reeves C, Carnoy M, Addy N (2013). Comparing opportunity to learn and student achievement gains in southern African primary schools: a new approach. International Journal of Educational Development.

[CR25] Roth McDuffie A, Graeber AO (2003). Institutional norms and policies that influence college mathematics professors in the process of changing to reform-based practices. School Science and Mathematics.

[CR26] Seymour, E. (2006), Testimony offered by Elaine Seymour, Ph.D., University of Colorado at Boulder, to the Research Subcommittee of the Committee on Science of the U.S. House of representatives hearing on undergraduate science, math and engineering education: what’s working? Wednesday, March 15, 2006.

[CR27] Seymour E, Hewitt NM (1997). Talking about leaving: why undergraduate leave the sciences.

[CR28] Spinath B, Stiensmeier-Pelster J (2003). Goal orientation and achievement: the role of ability self-concept and failure perception. Learning and Instruction.

[CR29] Stan Development Team. 2016. RStan: the R interface to Stan. R package version 2.14.1. http://mc-stan.org. Accessed 1 Jan 2017.

[CR30] Thompson PW, Castillo-Chavez C, Culbertson R, Flores A, Greeley R, Haag S (2007). Failing the future: problems of persistence and retention in science, technology, engineering, and mathematics majors at Arizona State University.

[CR31] Wach FS, Spengler M, Gottschling J, Spinath FM (2015). Sex differences in secondary school achievement––the contribution of self-perceived abilities and fear of failure. Learning and Instruction.

[CR32] Wagner JF, Speer NM, Rossa B (2007). Beyond mathematical content knowledge: a mathematician's knowledge needed for teaching an inquiry-oriented differential equations course. The Journal of Mathematical Behavior.

[CR33] Wu H (1999). The joy of lecturing-with a critique of the romantic tradition in education writing. How to teach mathematics.

[CR34] Yackel, E., Cobb, P., & Wood, T. (1991). Small-group interactions as a source of learning opportunities in second-grade mathematics. *Journal for Research in Mathematics Education*, *22*(5), 390–408.

[CR35] Yoshinobu S, Jones MG (2012). The coverage issue. PRIMUS.

[CR36] Zajacova A, Lynch SM, Espenshade TJ (2005). Self-efficacy, stress, and academic success in college. Research in Higher Education.

